# Effective Partnership Mechanisms: A Legacy of the Polio Eradication Initiative in India and Their Potential for Addressing Other Public Health Priorities

**DOI:** 10.4269/ajtmh.18-0938

**Published:** 2019-10

**Authors:** Jitendra Awale, Manojkumar Choudhary, Roma Solomon, Adesh Chaturvedi

**Affiliations:** 1 CORE Group Polio Project/India, Gurgaon, India;; 2 Public Health Expert, Jaipur, India

## Abstract

While many factors contributed to the successful elimination of polio from India, partnership and coordination mechanisms at multiple levels that have evolved over the years have been an important element. The lessons learned from these partnership and coordination mechanisms among various stakeholders involved in service delivery, surveillance, community mobilization, and governance deserve documentation as a legacy of the program. This article discusses the various processes and techniques adopted to build strong partnerships and coordination mechanisms among stakeholders by optimizing their strengths and using opportunities that lead toward the eradication of polio from India. Secondary data and literature review of relevant reports, papers and documents were adopted as the methodology for developing this research article. The article provides a model conceptual framework for partnerships and applies that framework to the CORE Group Polio Project (CGPP) partnerships in India and the partnerships among stakeholders for polio eradication in India. The learnings and expertise of the CGPP in developing, managing, and nurturing partnerships can be adapted and replicated for elimination or controlling other diseases (especially those that are vaccine-preventable as well as tuberculosis and vector-borne diseases) and for ending preventable child and maternal deaths.

## INTRODUCTION

Eradication of a disease that has plagued mankind from time immemorial is one of the greatest triumphs of public health. Polio is one of the very few diseases that have the potential to be eradicated because of the following: 1) the poliovirus affects only human beings and there is no animal reservoir, 2) the life span of the virus in the environment is very short, 3) immunity against polio is lifelong, and 4) an effective and low-cost vaccine is available. Nonetheless, the eradication of polio has turned out to be far more difficult than expected, requiring far greater sums of money, much broader stakeholder participation, and a much longer time span.^1^

**Figure 1.**
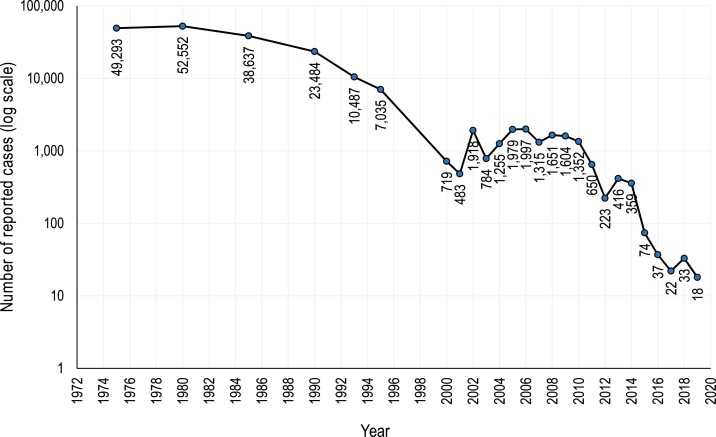
Number of poliomyelitis cases reported by year.^5^

In 1988, the World Health Assembly passed a resolution to eradicate polio. Since then, the Global Polio Eradication Initiative (GPEI), led by the WHO, the United Nations Children’s Fund (UNICEF), the US Centers for Disease Control and Prevention, and Rotary International, has provided support to governments of polio-affected countries. Other partners, including the U.S. Agency for International Development (USAID), the Bill & Melinda Gates Foundation, civil society organizations and non-governmental organizations (NGOs), and networks such as the CORE Group Polio Project (CGPP), have worked together to support the GPEI across the globe. Despite unanticipated challenges and setbacks, the goal of eradication has almost been achieved. The number of polio cases reported each year since 1971 has declined from 49,293 in 1971 (with the actual estimated number being 300,000) to 18 in 2019 as of May 7, 2019 (Figure 1).^2,3,5^ At present, wild poliovirus (WPV) transmission is limited to very small geographic areas with small populations within Afghanistan and Pakistan.^4^ The type 2 WPV was declared eradicated in 2015.^3^

This achievement has been attributed to the success of the polio eradication strategy, which consists of the following four pillars:

1. Strengthening of routine immunization
2. Surveillance for acute flaccid paralysis cases
3. Supplementary immunization activities†
4. “Mop-up campaigns”‡

**Figure 2.**
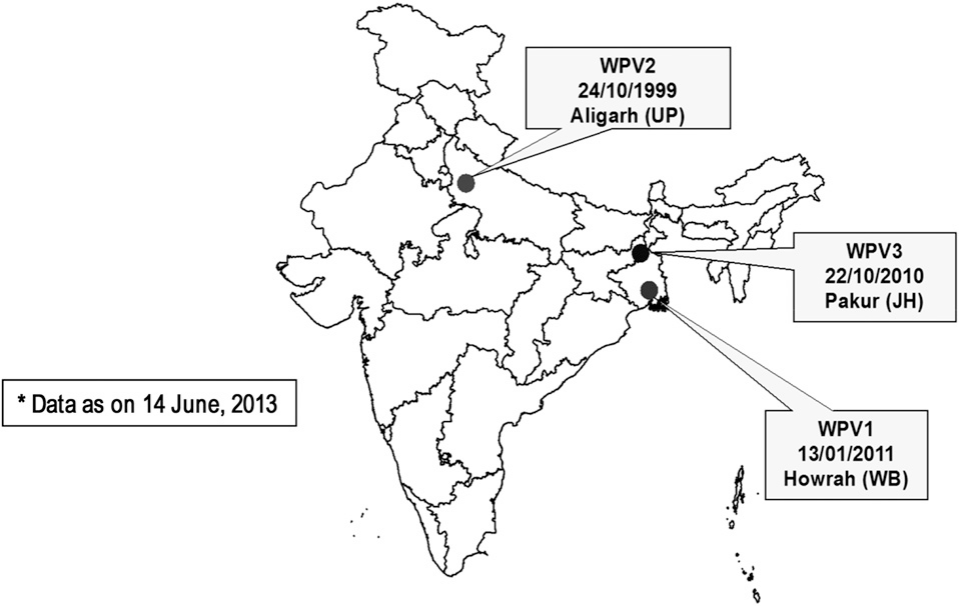
Last WPV cases in India by type and date. JH = Jharkhand; UP = Uttar Pradesh; WB = West Bengal.^2^

In 2002, India was one of the 10 countries known to have ongoing poliovirus transmission. India was characterized by areas with a high population density, low routine immunization coverage, suboptimal sanitation, and, therefore, intense indigenous WPV transmission.^6^ The last case of WPV was recorded in 2011 (Figure 2).^7^

At the 7th meeting of the South-East Asia Regional Certification Commission for Polio Eradication held in New Delhi on March 27, 2014, the region was declared polio-free, signifying that 80 percent of world’s population now lived in certified polio-free regions.^8–10^ This was the most significant milestone in the GPEI. Many experts believed that India was one of the toughest places to stop poliovirus transmission because of India’s high population density, poor sanitation, and low immunization coverage.^11^ Persistent efforts to reach and immunize every last child with oral polio vaccine (OPV) proved to be the key to India’s success. This required committed leadership, a devoted health workforce, tailor-made strategies, data-driven planning through surveillance and research, meticulous monitoring, targeted communication and social mobilization efforts, strong partnerships, and adequate funding. The remaining polio-endemic countries, Nigeria, Afghanistan, and Pakistan, have now adopted India’s best practices and lessons learned related to polio eradication. India’s polio program demonstrates that ambitious health goals can be reached even in areas with poor health systems, and India’s success now serves as a model for polio eradication and other public health programs around the world.^12^

As a longtime partner in the GPEI, the CGPP decided to document the partnership and coordination mechanisms which could be helpful to other public health initiatives. This documentation is also in line with one of the objectives of “Polio Endgame Strategy 2019–2023”^3^ and gives a clear road map to all the stakeholders for the last phase of the program. This plan is based on three themes: 1) eradication : stopping transmission; 2) integration: collaboration with immunization and emergency teams to eradicate polio and to protect populations; and 3) certification: certify and containment of all WPV cases and ensure long-term polio security.^13,14^ These themes underscore the need for a vital future global health policy and programs.

The partnership and collaboration model developed by the polio eradication program in India was the result of collective efforts of the government; UN agencies; NGOs at the international, national, and local levels; and other partners who worked tirelessly. The CGPP is one of the valuable partners in the national polio eradication program.§

Many times it has been said and heard that “partnerships” were key to the success of polio eradication in India as well as globally.^26^^–^^27^ However, this critical element of polio eradication has not been well documented. In particular, the role of civil society organizations such as the CGPP and the contribution of NGOs have been inadequately documented. One of the leaders of the GPEI has written that “much of the wealth of the GPEI’s lessons will never be captured in textbooks or academic articles because many of the details, especially with regard to what didn’t work, will survive only in the knowledge and experience of thousands of individuals who worked at various levels of this global initiative.”^15^ With this in mind, the CGPP has decided to document its experiences in India with partnerships as a key element in the success of the GPEI there.

This article discusses how the partnership for polio eradication evolved‖ and how the coordination mechanisms were institutionalized. We describe partnerships at two levels:

1. The CGPP partnership in which international and national NGOs collaborated together at the country level under the leadership of a secretariat to support polio eradication activities.
2. The partnership among governments (national and states), the WHO, UNICEF, Rotary International, and the CGPP.

## METHODS

The authors reviewed reports, articles, working papers, and research articles pertaining to partnerships in health programs in general and to the health partnerships developed for polio eradication in India. We created a general conceptual framework for partnerships and then applied that framework to the CGPP partnership in India and the partnership among stakeholders for polio eradication in India.

## RESULTS

### Conceptual framework of partnership.

Based on a review of the literature, the following definitions and frameworks were found to be the most appropriate for the partnerships formed for polio eradication. A partnership comprises organizations that have common goals and objectives and that combine resources to implement collective activities. Partnerships can prevent duplication of efforts, ensure synergy of resources, and augment the overall leadership within the country.^16^ The term “partnership” is generally used interchangeably with various other terms such as collaboration, alliance, coalition, network, interorganizational relationship, joint advocacy campaign, and taskforce.^17^ A “collaboration continuum” exists which places partnerships along a spectrum ranging from philanthropic, in which a charitable donor and recipient exchange resources focused on specific activities, to integrative, in which “the partners’ missions, people, and activities begin to merge into more collective action and organizational integration.”^18^

A continuum of organizational relationships can be described within a systems science framework, as shown in Table 1, derived from a systems analysis tool referred to as the intervention-level framework.^19^ Table 1 describes the continuum of three aspects of such organizational relationships: 1) the paradigm under which the relationships operate, 2) the goals of the relationships, and 3) the structure of the relationships.^20^

**Table 1:** Continuum of organizational relationships across levels in a system^20^

System level	One end of the continuum: weak relationships composed of interactions and engagements	The other end of the continuum: strong relationships composed of partnerships
Paradigm	Philanthropic to transactional	Transactional to integrative
	Simple or basic trust	Authentic trust
Goals	Peripheral to mission	Central to mission
	Minor strategic value	Major strategic value
	Knowledge exchange	Organizational influence
	Co-branding, cause-related marketing	Policy or program change
Structure (including loops and subsystems)	Low level of engagement, infrequent interaction	High level of engagement, intense interaction
	Small, often one-way exchange of resources	Major (usually two-way) exchange of resources
	Narrow scope of activities	Broad scope of activities
	Organizational independence	Shared governance/interdependence
	Simple management	Complex management

Coalitions have been recognized as particularly effective and efficient vehicles for identifying and prioritizing system-change strategies and for making the changes happen. Partnerships have been identified as playing a central role in enabling community coalitions and community-based health programs achieve system change and address health disparities.^20^

### Partnerships of the CGPP.

The CORE Group is a membership association of more than 100 U.S.-based international NGOs that strengthen local capacity on a global scale to measurably improve the health and well-being of children and women in developing countries.^21^ It achieves this through collaborative NGO action and learning. In 1999, the CORE Group received USAID funding for polio eradication, establishing the Polio Eradication Initiative Project that later became known as the CGPP.

The purpose of the CGPP is to engage NGOs in polio eradication mainly through social mobilization, community-based surveillance for acute flaccid paralysis (AFP), cross-border coordination, and related activities. The CGPP structure includes a secretariat in each country where it operates, CORE Group international NGO members, and their local NGO partners. The secretariat consists of a small team of “neutral” technical advisors (neutral in the sense that they are independent from any one implementing partner). The secretariat team facilitates communication, coordination, and transparent decision-making among all partners—unifying the community-level expertise of international NGOs and local NGOs with the benefit of international knowledge and strategies of the GPEI partners. The CGPP countries have successfully implemented the secretariat model to coordinate and promote civil society engagement in polio eradication, while simultaneously injecting a crucial community-level component through the coordinated activities of thousands of community health workers.^22^ The initial paper of this series provides a detailed history of the global CGPP and its secretariat model.^15^

As described elsewhere in this series,^15^ the CGPP began in 1999 at a critical juncture in the GPEI when it was becoming apparent that small pockets of unvaccinated children in a number of countries were the source of continued transmission of WPV and the traditional campaign approach to immunization, although achieving high levels of coverage, was not effective in wiping out these pockets of persistent transmission.

The CGPP secretariat in India works in close collaboration with the Ministry of Health and Family Welfare, the government of India, the WHO, UNICEF, Rotary International, and USAID. Until 2003, the CGPP and UNICEF had deployed social mobilizers in the areas where the polio program faced major challenges in terms of resistance to polio immunization. Most of the time, both agencies were working in the same geographic areas. After working together for a few years, the CGPP and UNICEF decided to form the Social Mobilization Network (SMNet) to provide more concentrated support for social mobilization in high-risk areas of Uttar Pradesh.^15^ During this time, the CGPP and UNICEF realigned and redistributed their work at the block (sub-district) level so that there was no duplication of efforts and resources.

The success of the CGPP arises from its ability to manage partnerships with diverse organizations that are working at various levels and that have very different capacities. The CGPP partnership, working since 1999 in India, could be one of the longest surviving international partnerships among public health programs in India. This partnership emerged as the outcome of working together and strong commitment of donors for an important shared goal. Initially, the CGPP did not have any ground rules, bylaws, formal guidelines, or legal standing to bind the partners together. The partnership evolved in the sense that the partners got better and better at working together over time despite the fact that no operational guidelines or terms of reference were available at the beginning. As the CGPP gained experience, a strong and trusting partnership emerged that was also quite flexible in its approaches. The partners of CGPP had different capabilities that were not necessarily related to polio or immunization. Some were good in community mobilization; others had expertise in program management, advocacy, implementation of targeted interventions, monitoring, developing demonstration sites, or implementing programs efficiently. The CGPP collaborated with diverse organizations that were locally recognized in their respective fields. The CGPP strategically positioned them to effectively address the goal of elimination of polio transmission. One size does not fit all, so for each partner, the CGPP secretariat had to use a different approach because their capacity and management structures were different. For instance, some of the local NGOs had simple management structures and was flexible in all forms of implementation. What they required from the CGPP mentoring on the technical aspects of polio eradication. On the other hand, some large organizations had complex, bureaucratic management systems that had to be simplified for speedy implementation in the field. Coordination with the local implementing NGO partners and with government required a different strategy. While working with the government, the CGPP secretariat represented a group of NGOs, with the government in a leadership position. While working with NGO partners, the secretariat provided coordination, technical expertise, and collaboration to build their capacity.

Similarly, to work with donors and partners (e.g., USAID, the Bill & Melinda Gates Foundation, the WHO, and UNICEF) required tailored approaches that had their own pace, involvement, and commitments. Fortunately, the CGPP secretariat and its partners learned from experience and started engaging effectively with these different categories of partners to coordinate and to channel resources. Four partnerships evolved over the life of the project^23^:

1. The CGPP/India working with USAID, the CORE Group, and CGPP global headquarters in Washington, DC
2. The CGPP/India Secretariat, coordinating the consortium of international, national, and local NGOs and other community-based organizations
3. The CGPP/India, working with the SMNet, UNICEF, the WHO National Polio Surveillance Project (NPSP), and Rotary International
4. The CGPP/India, working with the government of India

**Figure 3.**
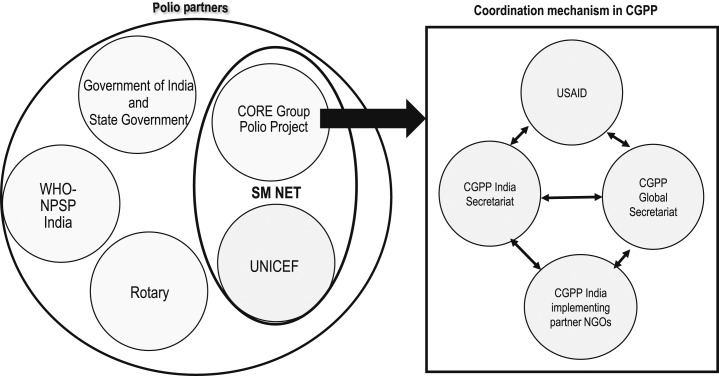
Polio partnership landscape in India.

The CGPP secretariat plays different roles while partnering with different agencies. Its secretariat coordinates with a consortium of international and national NGOs, provides technical support to them, and represents them at various levels. As a partner in the SMNet, the CGPP consortium partners with UNICEF in the field and with the WHO-NPSP, Rotary International, and the government at various levels. Figure 3 depicts the complex landscape of these partnerships.

In 1999, the CGPP established a secretariat in New Delhi to coordinate activities of the CGPP in India as well as in Bangladesh and Nepal. Five CORE Group international NGO members initially agreed to work directly or through their NGO partners: CARE, the Christian Children’s Fund (now Child Fund), Project Concern International (PCI), the Adventist Development and Relief Agency (ADRA), and World Vision. At present, three international NGOs are working with the CGPP in India: PCI, ADRA, and Catholic Relief Services (CRS) India. They and their eight local NGO partners work in 12 districts of Uttar Pradesh, covering a population of approximately 30 million people. Thus, international and national/local NGO partners willingly started working together under the leadership of the CGPP independent secretariat.^23^ The CGPP in India complemented the efforts of the Ministry of Health and Family Welfare, the NPSP, the WHO, and UNICEF in high-risk areas as per their mutually agreed strategy.

Soon the CGPP secretariat became the critical nexus between those working in these high-risk areas (namely, NGOs and the local Ministry of Health and Family Welfare staff) and the high-level technical and donor entities (the U.S. CDC, USAID, the Bill & Melinda Gates Foundation, the Ministry of Health and Family Welfare, the government India, the NPSP, the WHO, and UNICEF).

The CGPP secretariat, as well as UNICEF, the NPSP, and Rotary International, transferred the strategies and implementing activities developed at a higher level down to the local field level through their respective organizational channels while at the same time communicating grassroots-level voices to policymakers. The CGPP secretariat also provided technical support to its collaborating NGOs.

The CGPP secretariat in India convened quarterly meetings of its implementing partners, including field staff, to review its activities and plans. The secretariat staff made regular field visits, which were useful for supportive supervision and for providing on-the-job training to the field staff. These field visits were also valuable for strengthening coordination with district-level officials of the government and other partners such as the WHO, UNICEF, and Rotary International, all of whom had staff stationed in the area.

Every year, the CGPP secretariat in India organized training for all of the NGO staff using master trainers who were handpicked from among the partner NGO staff and then trained by the CGPP. These trainings were effective at building capacity and strengthening coordination.

### Partnerships of the CGPP with the government of India and its states, the WHO, UNICEF, and Rotary International.

The partnership between various stakeholders was achieved using multiple mechanisms such as the Social Mobilization Working Group (described further in the following paragraphs) and coordination meetings among partners at the sub-national and district levels. The Social Mobilization Working Group consisted of representatives from UNICEF, the WHO-NPSP, the CGPP, Rotary International, and the government of India. This group frequently discussed the communication and social mobilization challenges of the polio eradication program and suggested necessary changes in the strategy.

The role of each partner was well defined and distinct to ensure complementarity and to avoid duplication of effort. The government of India provided leadership as well as human, material, and financial resources, whereas the NPSP supervised the surveillance of acute flaccid paralysis, provided technical support for the operation of the polio immunization campaigns (called Supplemental Immunization Activities), and supported the monitoring of the supplemental immunization activities. Rotary International played a very important role by supporting advocacy and also providing financial resources. UNICEF provided technical support for vaccine procurement and media campaigns at the national and state levels, and social mobilization through the SMNet. The CGPP in India supported social mobilization through the SMNet; represented NGOs at the national and state levels for better coordination, facilitation, and support; and also provided essential technical and supervisory support to the NGOs.

**Figure 4.**
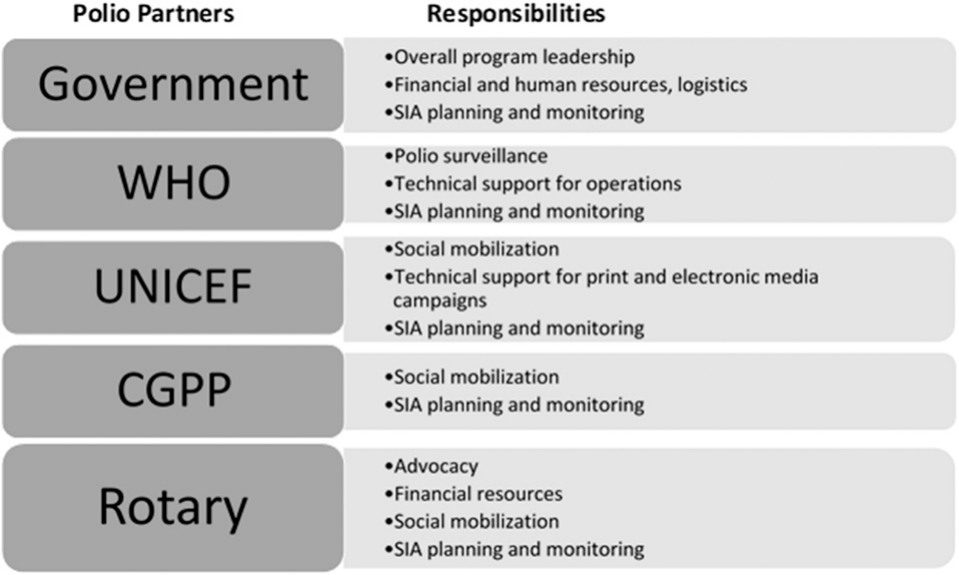
India polio partners and their responsibilities for the SMNet in Uttar Pradesh.

UNICEF, the NPSP, Rotary International, and the CGPP formed the SMNet in 2003 in the state of Uttar Pradesh, and their respective roles are outlined in Figure 4. This resulted in the inclusion of NGOs in a vital decision-making and polio program-implementation platform. This large collaborative network of NGOs also developed the capacity to support other national, regional, and community disease-control initiatives and routine immunization. This role division is summarized in Figure 4.

**Figure 5.**
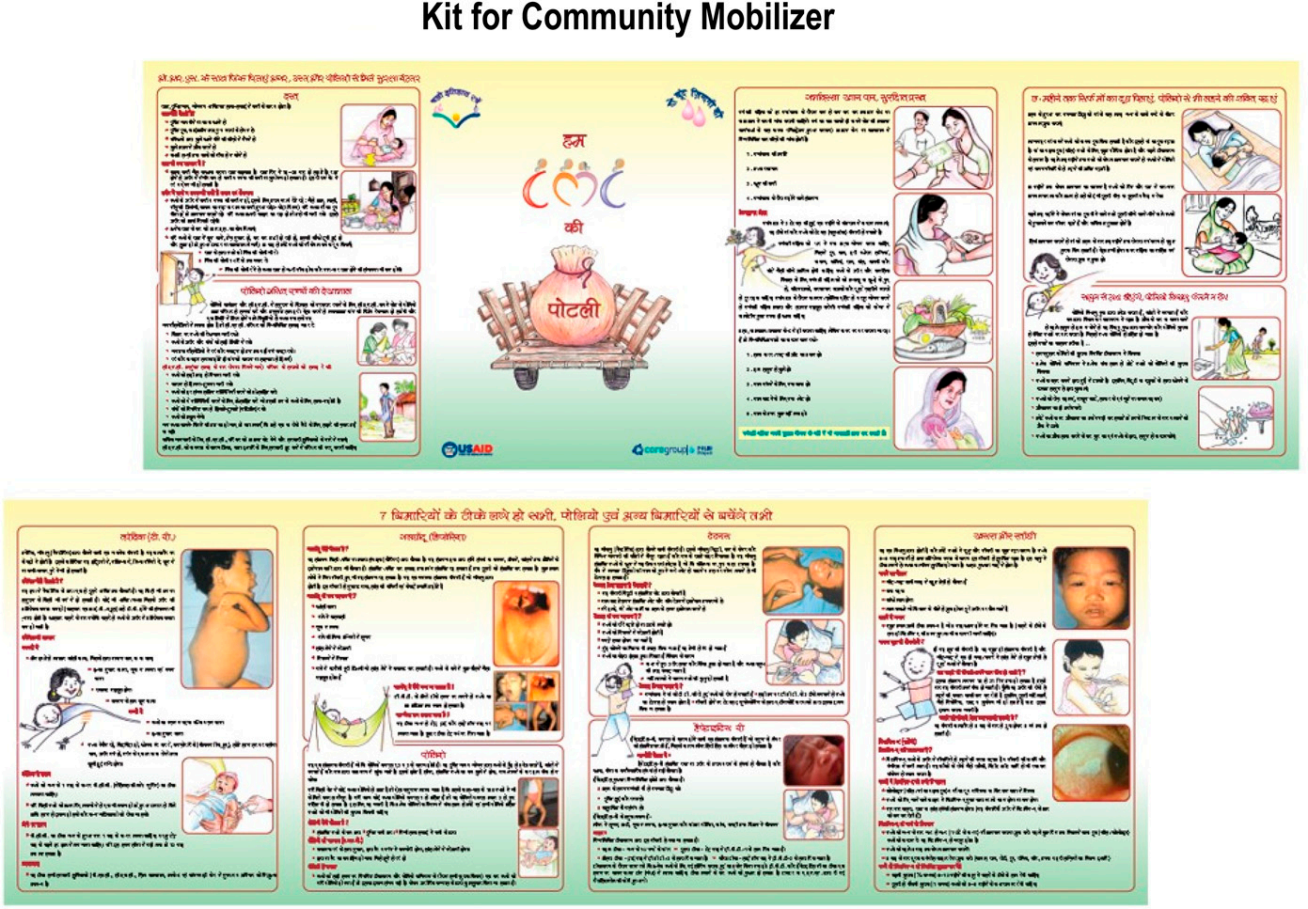
Sample training materials of the CGPP.

**Figure 6.**
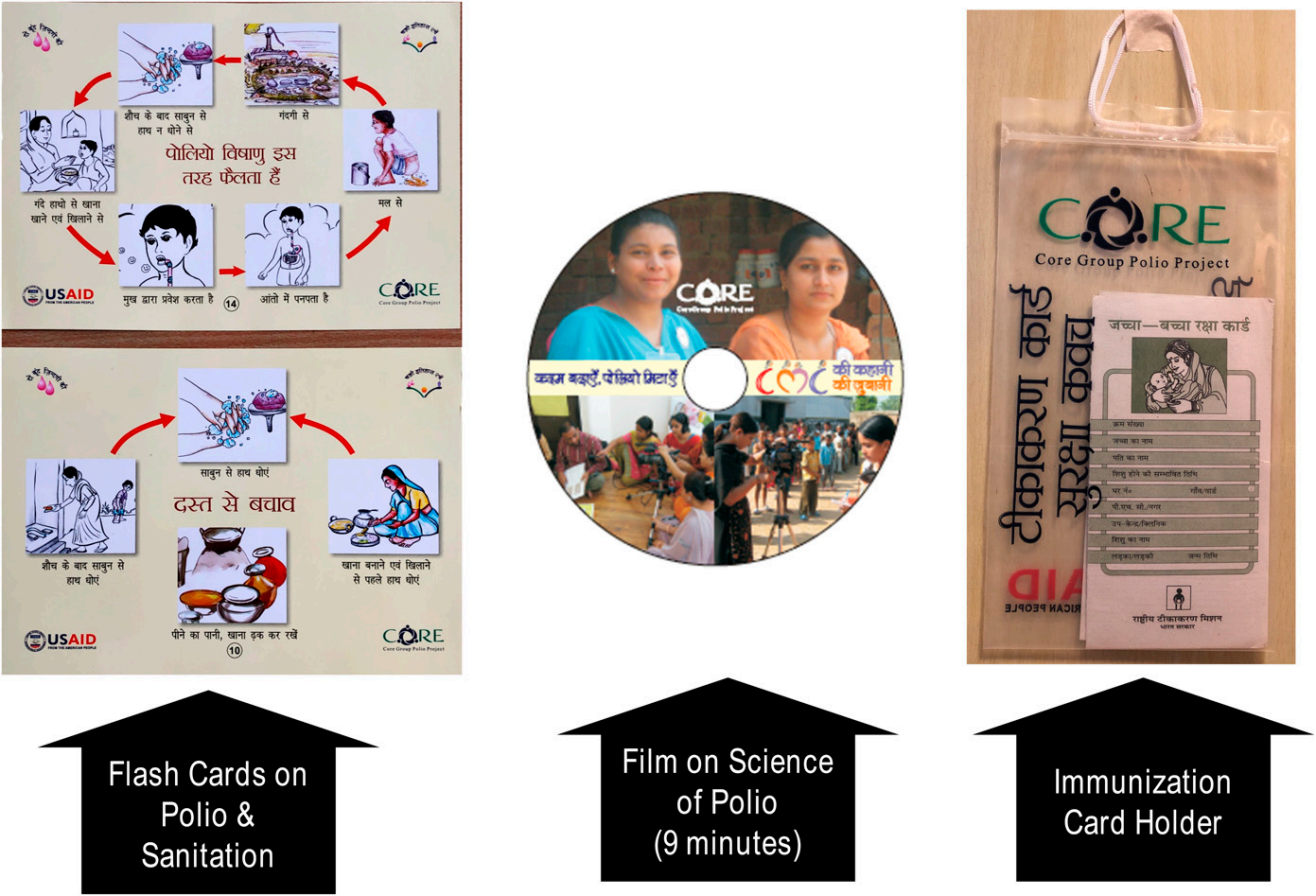
Additional sample training materials of the CGPP.

The CGPP and UNICEF developed various materials for training, behavior-change communication, supervision, and human resource management with uniform guidelines. Examples of these tools are provided in Figures 5 and 6.

These materials were used consistently across the SMNet areas. Gradually, the SMNet standardized field staff positions and functions, expanded and refined data collection, and incorporated increasingly focused behavior-change communication techniques. CORE Group Polio Project and UNICEF implemented synchronized social mobilization activities using community-level workers called community mobilization coordinators. The three-tier network of community mobilizers (at the community, block/subdistrict, and district levels) carries out the main work of the SMNet. Community mobilization coordinators work with underserved communities in planning, implementing, and monitoring social mobilization and other immunization activities in high-risk areas.^10,24^

The purpose of partner meetings was to sort out operational issues and update all the concerned stakeholders about the current field situation. For smooth and effective coordination, mechanisms were created to address the implementing challenges. These structures were made functional through frequent engagements, such as meetings, field visits, and task force meetings held by the CGPP secretariat and other partners.

At the national level, representatives of the WHO, the NPSP, UNICEF, the CGPP, and the government of India formed a Social Mobilization Working Group. The India Expert Advisory Group for Polio, represented by independent experts, government functionaries, academicians, and polio partners, met every 6 months to review and guide the program at the national level. State task force meetings were also implemented at the state level in Lucknow, the capital of Uttar Pradesh. For these meetings, government and other polio partners came together to deliberate and decide on program priorities and issues to address.

Under these structures, a district task force was formed under the chairmanship of the district collector and magistrate, a government official who is the key decision-maker at the district level. Either the district collector and magistrate (the senior most government official) or the district chief medical officer chaired its meetings to review the progress of polio eradication in the district. The meeting was attended by senior officials of all the relevant government departments and representatives of the CGPP, UNICEF, Rotary International, religious leaders, and the NPSP to review the vaccination coverage and identify administrative, operational, and communication barriers encountered during the previous polio vaccination round. As a result of these meetings, it was possible to address gaps and barriers on a timely basis.

The district task force meeting was the forum for discussing evidence-based actions and next steps. Accordingly, the district collector and magistrate issued directives. Reaching every child, whether in the house, outside the house, or even out of the village/city at the market place or on the move at migrant/nomadic sites, required support from different departments of the government and from the community. Block task forces were formed at the sub-district level. A block is the smallest administrative unit of the government administrative structure. Block review meetings (also called sub-district-level meetings) were conducted regularly and were attended by district and block level officials of WHO, UNICEF, and CGPP partners.

**Figure 7.**
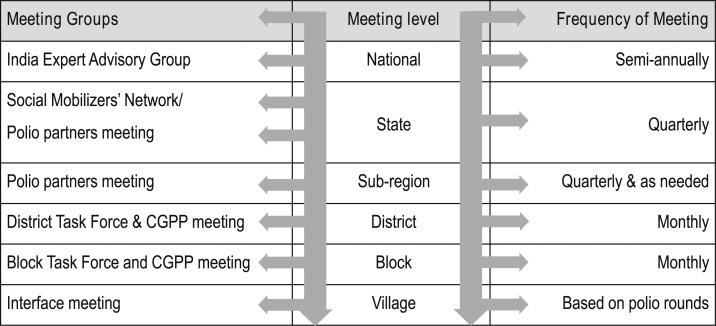
Structure and frequency of meetings of the Social Mobilization Working Group.^11^

Village-level Social Mobilization Working Groups and interface meetings produced important functional partnerships and strong coordination among frontline workers (village or urban ward-level volunteers, community mobilization coordinators, vaccinators, and community influencers). Block-level supervisors from the SMNet and the NPSP also attended these “interface” meetings. These meetings helped to mobilize local resources to address local issues in a timely fashion. Figure 7 describes the institutional structures and the frequency of the group meetings.^11^

Participation of CGPP representatives in these meetings helped to improve networking and coordination with all partners. This led to improved service delivery to the community. One example is the facilitation of health camps/routine immunization drives in selected outreach areas where resistance was high. These were a response to the frequently stated request for better basic health services. These regular meetings and the subsequent health camps led to improved relationships between the health service providers such as immunization workers and the community.

### Challenges faced by the CGPP.

Success can only be achieved once challenges have been mitigated. The CGPP also faced several such challenges. Because there were many diverse partners and some were large international organizations (such as the WHO and UNICEF), whereas others were local, small community-based NGOs that did not have much experience with this type of collaboration, there was apprehension and even mistrust between these groups of organizations. The larger partners had doubts about the capacities of NGOs, whereas the NGOs were apprehensive about losing their identity and operational freedom working under a big umbrella. Here, the CGPP secretariat played a facilitating role between them to develop mutual trust and clarify doubts so that good teamwork could be established.

Enabling partner NGOs to deliver in an emergency mode as part of a large-scale program while overcoming bureaucratic hurdles was a major challenge. Most of the NGOs did not have the experience of working very rapidly and rigorously, for example. In the first few years of this collaboration, supplemental immunization activity (SIA) rounds were conducted every month. This required a lot of work, and some NGOs were not used to this type of intensive, almost continuous campaign work. The CGPP facilitated and invested resources in building the capacity of these NGOs. Working with UNICEF, the CGPP strengthened the SMNet by, as mentioned earlier, institutionalizing uniform nomenclature and terms of reference for workers/staff and their training. This took a lot of effort, as each NGO had its own organizational hierarchy, salary structure, mandate, and priorities.

 The changing epidemiology of local poliovirus transmission required considerable operational flexibility and preparedness. The community considered the SMNet staff as the face of all government machinery. The community had apprehensions about the government’s programs and its functionaries. This made working as a bridge between the community and the government a major challenge. Following the strict requirements of the donor to focus all resources exclusively on polio was also challenging because of the great need (and community demand) to expand the focus to other problems beyond polio that were much more important to the community. However, the CGPP and SMNet succeeded in addressing these challenges through constant effort, facilitation, understanding of the complexities at the ground level, and establishment of effective and regular communication between the community, NGOs, government, and donors.

**Figure 8.**
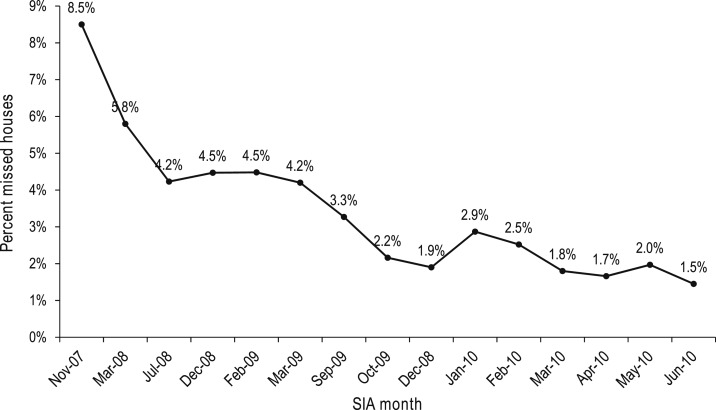
The percentage of missed households at the time of supplemental immunization activities for polio in the SMNet catchment areas of Uttar Pradesh, 2007–2010.

**Figure 9.**
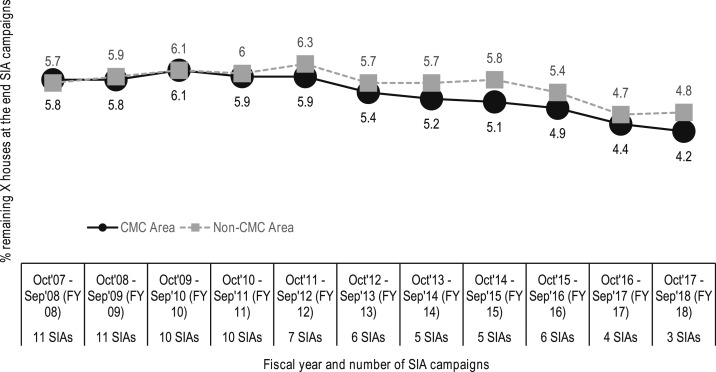
Trends in the percentage of missed houses during SIA campaigns in the districts covered by the CGPP/India, 2008–2018.^25^

Figure 8 shows the downward trend in the percentage of missed households that were attributable to resistance that occurred after involvement of SMNet mobilizers began. This is itself strong evidence of the effectiveness of the partnerships for polio eradication in India. Figure 9 shows the percentage of missed houses in the districts covered by the CGPP, comparing the outcomes for those districts with community mobilization coordinators with those where community mobilization coordinators were not working.^25^

## DISCUSSION

The success of the SMNet in contributing to the elimination of WPV transmission in India is evidence that the secretariat model was effective in enabling international, national, and local NGOs to develop strong long-term partnerships in which mutual respect emerged and in which each organization recognized that all participating organizations were complementing the capacities of the others. Field-based learning and continuous analysis of inputs, processes, and outputs were responsible for the emergence of such strong partnerships. The secretariat model enhanced the partnerships because it facilitated NGO representation in the immunization task force at state and national levels. The secretariat represented all consortium members as a single entity to the government and other international agencies such as the WHO and UNICEF. This also helped the government and polio partners in the sense that instead of dealing with many NGO organizations they had to deal with only one.

The CGPP proved to be a good example of a consortium of NGOs coordinated by a secretariat that is not directly involved in implementation. Because of its neutral leadership (in the sense that it was independent of the NGOs themselves), the CGPP secretariat could provide effective coordination with the government at various levels and with UN agencies, and it could monitor program implementation of multiple partners in an unbiased manner. There was ample evidence that the secretariat-led model resulted in clear and concrete benefits to the program and its partners.

However, the secretariat model does need some improvement for future replication for purposes beyond polio eradication or for scaling up in other geographic regions. The CGPP secretariat has no formal independent legal identity of its own; neither does it have a memorandum of understanding with the government. The lack of legal status acted as a barrier during negotiations at all levels. A secretariat that is coordinating the efforts of international NGOs, national and state government officials, and multinational organizations needs to have a distinct legal status and authority in financial and human resource matters. In addition, a lack of an operations manual or working guidelines led at times to some confusion in roles and responsibilities.

As the GPEI nears its goal of polio eradication, now is the right time to obtain legal registration for the CGPP secretariat in India. This would help to sustain the brand value of the CGPP and its relationships with the community and the government. It would also enable the secretariat to address other public health issues in India. A small group of advisors would also help it to further its agenda in the country.

Box 1 **Challenges overcome by the CGPP^20,25^**

**LEARNING TO MANAGE CHANGE**

During the course of implementation over the previous 18 years, various changes in the CGPP partners have taken place. In 2003, the CRS joined the CGPP in new areas. In 2005, there was a reassignment of districts between the CRS and the ADRA. In 2007, World Vision withdrew, and its areas were assigned to the PCI and the local NGO partners of the PCI. All these changes happened at a time when the polio eradication program was facing multiple challenges, such as a large number of families refusing vaccination and an increasing number of polio cases. The WHO, UNICEF, and the government all shared their apprehensions then that these changes might hamper social mobilization in the CGPP areas. But the CGPP secretariat and CGPP partners did manage these transitions very effectively without any discernible effect on program implementation. This was achieved by a very proactive secretariat team at all levels and capacity building of field staff. The result was that an NGO partner was able to join the CGPP at various stages of the CGPP implementation and begin its own involvement at the stage where the CGPP was at the time.

There was a need for considerable compromise and adjustment when the CGPP partnership with its consortium members was first established. Problems that arose because of ideological and strategic differences had to be sorted out. Another indicator of success of this partnership model was that changes in leadership and staff turnover did not affect the functioning or the effectiveness of the CGPP. These partnerships and networking have been going on for 20 years now and the SMNet has been functioning smoothly for 16 years. The feasibility of the partnership was facilitated by the fact that the partners were dealing with a global initiative to eradicate polio, which called for united, synergistic action.

A large set of diverse partners (international and national/local NGOs, UNICEF, Rotary International, the WHO, the CORE Group, and the government) all worked together for eradicating the poliovirus from India. This partnership that has been sustained for two decades now proves that simple mechanisms developed by the partners could be major lessons for global public health initiatives that require collaborative functioning.

The partners had a common vision, mission, and program goal. This singular focus on polio eradication brought about cohesion of forces despite the diversity of the partners. There was a sense of ownership resulting from involvement of all the partners at every stage of the program. There was minimal conflict of interests because of a clear overarching goal of global importance, a uniform agenda, role clarity, and the operational freedom with which partners had to continue with their other individual agendas apart from polio eradication.

A high level of enthusiasm between all the partners could be sustained for a long period of time because of the shared goal of polio eradication. All the partners were willing to seize the opportunity and be part of the global initiative. There was considerable pride in their involvement in the cause of polio eradication. Motivation was sustained because of the measurable reduction in polio cases.

The CGPP secretariat and consortium model ensured transparency regarding the structure of the partnership and the participatory nature of decision-making. The model was also able to establish accountability resulting from participatory monitoring and peer influence. The Polio Eradication Program in India was open to scrutiny by many groups, including the government and international agencies. This was one of the programs that used monitoring data and took observations of the monitors very seriously. Every evening during SIAs, senior functionaries from the government and partners reviewed the performance at all levels and immediate corrective actions were taken by the concerned partners. If there were unresolved communication challenges or if a few families or group of families were refusing to vaccinate their children in a specific area (such as a village or an urban area), then immediately a joint action plan would be made with the CGPP/UNICEF, government, and WHO officials to resolve the issues and vaccinate each and every child. This helped to establish accountability. Characteristics of successful partnerships with communities identified by Shortell et al. and examples of these successful partnership characteristics of the CGPP are summarized in Table 2.^26^

**Table 2:** Examples of successful partnership characteristics for the CGPP in India

Successful partnership characteristics identified by Shortell et al.^26^	Examples from the CGPP
Manage size and diversity	Established formal working relationships among a diverse set of international, national and local NGOs, United Nations Children’s Fund, the WHO, Rotary International, and the government working in different areas/districts
	Used work groups
	Set goals
Use multiple components of leadership	Established the CGPP secretariat as an independent entity to coordinate NGO partners
	Recruited senior government officials to lead all national, state, district, and subdistrict-level task force meetings that were supported by partners
	Recruited members from the community to assist
	Used respected community leaders to reach out to needed partners
Maintain focus	Applied a high-risk approach to focus on prioritization of resources
	Restricted partner organizations to those serving identified program needs
	Identified a designated point person for each partner organization
	Established work groups around specific needs (such as social mobilization)
Manage and channel conflict	Organized partner meetings at various levels to discuss issues, enhance communication, and improve problem solving
	Undertook joint activities such as joint review meetings to develop a common understanding among partners and to establish better coordination
	Adjusted organization policies to meet program challenges
	Used real-time monitoring and evaluation data to guide decision making
Recognize program life cycles and the need for “succession planning”	Developed a standard orientation for new partners and for new staff of existing partners
	Redistributed geographic areas of responsibility (joining of the new partners or areas at various stages of the program did not affect the pace of the program implementation)
	Anticipated and planned for crisis management (such as the preparation of a rapid response team).
“Patch” or reposition assets	Used independent monitoring and surveillance data to allocate areas to partners and resources

CGPP = CORE Group Polio Project; NGO = non-governmental organization.

With respect to “patching” and repositioning of assets, it is of particular note that in the beginning, the CGPP was working in many states in India but as program needs changed along with resource constraints, the work areas narrowed to only Uttar Pradesh state. In 2011, a WPV case was identified in Hawrah district in West Bengal. As an emergency response to this, the CGPP established a new network with three local NGO partners and funding support from UNICEF. A team of experienced CGPP staff from Uttar Pradesh were deployed on a rotation basis for field support to local teams.

## CONCLUSION

The partnership model developed by the Polio Eradication Program in India was highly functional and played a crucial role in eliminating polio from the country. The CGPP was itself an important partnership that linked NGOs with district- and block-level partners in high-risk areas. The shared goal of polio eradication made it possible for the partnership model to be effective. The success of polio eradication has strengthened the motivation of partners to work together and collaborate for addressing public health issues.

These coordination mechanisms are now being used for strengthening routine immunization and introducing new vaccines to India. Similar coordinating structures such as the Immunization Action Group (IAG) at the national level, and immunization task forces are being formed at state, district, and subdistrict levels for strengthening routine immunization and introducing new vaccines such as rubella, and rotavirus vaccines. Members of the IAG are similar to those of the India Expert Advisory Group (IEAG) for polio, with few additions. Current members and invitees for the IAG are representatives of the Ministry of Health and Family Welfare, the government of India, UNICEF, the WHO, USAID, the Bill & Melinda Gates Foundation, the CORE Group, the Immunization Technical Support Unit, John Snow, Inc., the Indian Council of Medical Research, the United Nations Development Program, and GAVI, the Vaccine Alliance.^27^

The state task force on routine immunization now works in a similar fashion as do the state task forces for polio. In Uttar Pradesh state, where polio partners have focused their efforts, they have also continued their contributions to strengthening routine immunization. The state task force on routine immunization continues to meet frequently to discuss progress and challenges. Special routine immunization campaigns such as Mission Indradhanush have adopted a similar approach for coordination that was used for polio immunization campaigns.

The CGPP in India has traveled a long journey and encountered multiple challenges, but it found ways of overcoming these challenges. Developing partnerships has proven to be a critical piece of the polio elimination success in India along with social mobilization and communication interventions for polio immunization campaigns, capacity building of functionaries, and monitoring. The government of India and all partners need to continue their efforts to sustain this partnership for addressing other public health priorities.

The lessons learned and expertise gained by the CGPP in developing, managing, and nurturing partnerships can be adapted and replicated to control other diseases (for vaccine-preventable diseases as well as for tuberculosis and malaria) and for elimination of preventable child and maternal deaths. Maintaining and further strengthening the partnership model for polio eradication in India and using it to address other public health priorities in India could be the real legacy of the Polio Eradication Program in India.

## Footnotes

† These were often monthly “rounds” in which all children were given a supplementary dose of polio vaccine.

‡ Mop-up campaigns are door-to-door immunizations that are carried out in specific areas where the virus is known or suspected to still be circulating.

§ The CGPP is a consortium of NGOs currently working on polio eradication in eight affected countries: Nigeria, Ethiopia, South Sudan, Kenya, Somalia, India, Afghanistan and Uganda. The CGPP successfully graduated from Bangladesh, Nepal, and Angola after cessation of wild poliovirus transmission there.

‖ The term “evolved partnership” refers to the fact that the ability of partners to work together improved over time. No operational guidelines or terms of reference were available when the partners initiated their collaboration.
